# Cellular Congenital Mesoblastic Nephroma in a Newborn

**DOI:** 10.21699/jns.v6i2.564

**Published:** 2017-04-15

**Authors:** Prasanta Kumar Tripathy, Susmita Behera, Hiranya K. Mohanty

**Affiliations:** 1 Department of Pediatric Surgery, SCB Medical College, Cuttack, Odisha, India; 2 Department of Pathology, SCB Medical College, Cuttack, Odisha, India

A 7-day-old male neonate was presented with abdominal distension and vomiting. There was a large right sided renal mass crossing 2 cm beyond the midline. CT scan of abdomen suggested a heterogenous tumor arising from the right kidney. Right radical nephroureterectomy was performed and histopathological diagnosis of congenital mesoblastic nephroma, cellular variety was made. Patient is doing fine on follow-up (2years).

Congenital mesoblastic nephroma(CMN) is a rare stromal neoplasm of kidney. CMN typically affects neonates and young infants. The most common presentation of CMN is asymptomatic abdominal mass [1]. Hypertension can occur due to increased rennin levels as a result of trapped glomeruli in the tumor [2]. A probable diagnosis can be made by the imaging characteristics, but histological study is the definitive test for the diagnosis. CMN occurs in two histological subtypes. Classical variant (24%) represents benign disease and cellular variant (66%) represents aggressive pathology. There is a mixed variant (10%) having features of both type [2]. The classical variety usually occur in infants below 3 month of age. Grossly it appears as solid, firm and non-encapsulated mass with irregular margin. Histologically it consists of interlacing fascicles of fibroblastic cells with thin tapered nuclei, pink cytoplasm, low mitotic activity and abundant collagen deposition [3]. Cellular varieties have larger areas of haemorrhage with cystic and necrotic components. Histologically, there are poorly formed fascicles which give a sheet like growth pattern. The tumor shows high mitotic rate figures [1]. They usually invade perinephric fat and connective tissue [2]. However, histological diagnosis of mesoblastic nephroma is a challenge. There are chances of misdiagnosis because of resemblance with other common kidney tumors and lack of exposure to this rare pathology. So authors suspect it is an under-reported pediatric neoplasm [4]. Radical nephroureterectomy is the mainstay of treatment in CMN. Chemotherapy should be given in case of incomplete removal or tumor rupture during surgery. Local recurrence and metastasis have been reported in about 5% of cases of CMN [2,3], usually within one year following surgery. Although classical variety of CMN is much more common as compared to cellular type under 3 month of age, this case was found to be of cellular variety. 

## Footnotes

**Source of Support:** None

**Conflict of Interest:** None

## Figures and Tables

**Figure 1:**
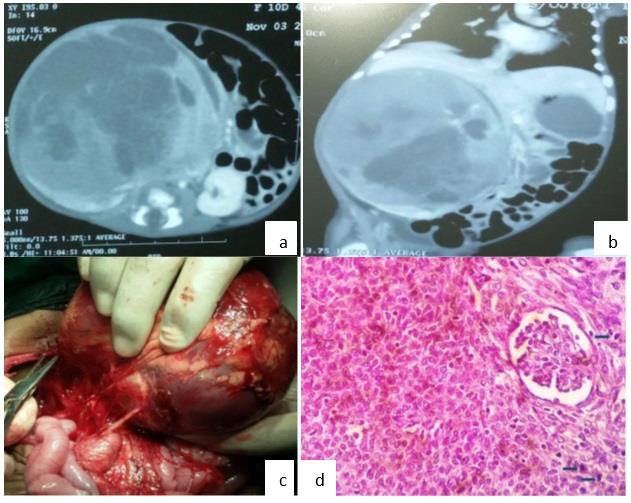
(a) Computed tomography(CT) abdomen - axial image showing large tumor, heterogeneously enhancing with areas of necrosis arising from right kidney displacing vessels and crossing midline. The left kidney is normal. (b) Coronal image showing large renal mass compressing the liver (c) Right side Radical nephrectomy- Intraoperative picture (d) Histology specimen suggesting congenital mesoblastic nephroma, cellular type entrapping the normal glomerulus of kidney. Arrows showing the numerous mitotic figures.
